# Ultrastructural pathological changes in the cochlear cells of connexin 26 conditional knockout mice

**DOI:** 10.3892/mmr.2013.1614

**Published:** 2013-08-05

**Authors:** LAN LIN, YUN-FENG WANG, SHU-YI WANG, SHAO-FENG LIU, ZHANG YU, LIN XI, HUA-WEI LI

**Affiliations:** 1 Department of Pathology, Eye and ENT Hospital of Fudan University, Shanghai 200031, P.R. China; 2 Department of Otolaryngology, Eye and ENT Hospital of Fudan University, Shanghai 200031, P.R. China; 3 Department of Electron Microscopy, Medical School of Fudan University, Shanghai 200031, P.R. China; 4 Department of Otolaryngology, Emory University School of Medicine, Atlanta, GA 30322, USA

**Keywords:** ultrastructural morphology, connexin 26, gene knockout, cochlea

## Abstract

Mutations in the gene of connexin 26 (Cx26) are the most common cause of human non-syndromic hereditary deafness. The pathogenesis of deafness caused by Cx26 remains uncertain. To explore the basic mechanism underlying Cx26 null mutations, ultrastructural changes and a number of marker proteins in the cochlear sensory epithelium of Cx26 conditional knockout mice were observed in the current study. Cochlear specimens were obtained from Cx26 conditional knockout mice (cCx26ko), while wild-type mice served as controls. Antibodies against the pillar cell marker P75, the supporting cell marker prox1 and hair cell markers myosin 6 and phalloidin were labeled in different cells of the cochlear sensory epithelium of cochlear cryosections. The ultrastructural morphology of cochlear sensory epithelium was observed using transmission electron microscopy. Following the observation of cochlear sensory epithelium cell markers for hair cells and supporting cells, no significant changes were observed at the early stage, while the tunnel of the organ of Corti and Nuel’s space was not developed prior to hearing onset in cCx26 knockout mice. Cell death was observed from postnatal day 10 (P10). The only region of surviving cells observed in the cochlea was the Hensen cell region, where microglia-like cells appeared following P180. Overall, the present study showed an abnormal ultrastructural morphology in the cochlear sensory epithelium in cCx26ko mice. Microglia-like cells may be involved in the process of cell degeneration in cCx26ko mice.

## Introduction

Congenital deafness is one of the most common human genetic birth defects, occurring in 1 or 2 of every 1,000 births. Connexins (Cxs) are membrane proteins that form intercellular channels known as gap junctions (GJs). Six Cx subunits form a connexon (hemichannel). Two hemichannels in adjacent cells align to form a complete GJ allowing the exchange of ions and molecules with a molecular weight <1,000 Da (1). Genetic linkage and mouse genomic studies have demonstrated that normal functions of Cx26 are essential for hearing, although the mechanisms underlying deafness caused by Cx mutations remain unclear. A number of subtypes of Cxs are reported to be expressed in the mammalian inner ear, with Cx26 being one of the most predominant (2). Previous studies found expression of Cx26 in the stria vascularis, spiral ligament, spiral limbus and supporting cells of the human cochlea (3) Among >100 deafness genes identified thus far, mutations in the gap junction protein Cx26, coded the by *GJB2* gene, account for more than half of hereditary non-syndromic deafness in humans (4,5).

Loss of Cx26 is hypothesized to prevent recycling of K^+^ following sound stimulation, with elevated K^+^ in the extracellular perilymph inhibiting uptake of the neurotransmitter glutamate, which ultimately results in cell death. Generating Cx26 mutant mouse models has been crucial in understanding deafness mechanisms. Complete knockout of Cx26 in mice results in neonatal lethality, preventing examination of its function in the adult inner ear (6). Cohen-Salmon *et al*(7) performed targeted ablation of Cx26 in the epithelial gap junction network in the cochlea using otogelin-driven Cre expression. In this previous study, deafness in the mutant mice was reported to be the result of cell death in the organ of Corti beginning at postnatal day 14 (P14), soon after the onset of hearing, which is ~P13 in mice. The initial site of cell death is found near the inner hair cells, consistent with the K^+^ accumulation hypothesis. Other conditional Cx26 knockout mouse models have been developed. Kudo *et al*(8) established a mouse model expressing the dominant-negative Cx26 mutant R75W in the inner ear. Wang *et al*(9) generated three different lines of conditional mouse models. Cx26 mutant mice from the Kudo *et al* and Wang *et al* studies developed histological abnormalities prior to P14, which is in contrast to the initial report from Cohen-Salmon *et al*(7).

Since different phenotypes are reported for different conditional Cx26 knockout mouse models, the pathological mechanisms underlying deafness caused by Cx26 mutations remain unclear. Pathological changes in the organ of Corti observed at the ultrastructural level in Cx26 mutant mice are particularly lacking. The aim of the current study was to examine and document ultrastructural pathological changes of cochlear cells in previously generated Cx26 conditional knockout (cCx26ko) mice (9).

## Materials and methods

### cCx26ko mice

The cCx26ko mice were provided by Xi Lin at Emory University, Atlanta, Georgia. Data presented previously demonstrated that the hearing of cCx26ko mice is severely impaired (9). Detailed descriptions of the hearing of cCx26 mutant mice and light microscopy of the morphology of their cochlea have been published (9,10). The following experimental groups of cCx26ko mice were observed in the current study (two animals/time point): P8, P10, P18, P30, P60, P90, P120, P180 and one cCx26ko mouse aged 360 days. The control groups were two littermate-controlled wild-type mice at P10, P18, P30 and P360. The study protocol was approved by the Institutional Animal Care and Use Committee of Emory Univerity, Atlanta, GA, USA (protocol no. 255-2009).

### Immunostaining

Cochlear tissue was dissected using microdissecting tools under a stereomicroscope and fixed in 4% paraformaldehyde in PBS (pH 7.4) overnight at 4°C. Tissues were embedded in 10% gelatin dissolved in water for <2 h at room temperature, cut into small blocks (<3-mm cubes) and dehydrated by submerging in 2.3 M sucrose solution overnight at 4°C in an Eppendorf tube fixed on an orbital rotor. Cochlear cryosections of 8 μm were prepared (model CM1900; Leica Microsystems, Bannockburn, IL, USA). Antibodies against pillar cell marker P75 (11) (1:200 dilution) and the supporting cell marker prox1 (12) (1:800 dilution) were obtained from Chemicon (Temecula, CA, USA). Hair cell markers myosin 6 and phalloidin were labeled with antibodies from Proteus Bioscience (Ramona, CA, USA) and Sigma-Aldrich (St. Louis, MO, USA). The secondary antibody used was donkey anti-mouse conjugated to rhodamine (1:200 dilution, Jackson ImmunoResearch Lab. Inc., West Grove, PA, USA) or goat anti-rabbit IgG conjugated to Alexa Fluor 488 (Jackson ImmunoResearch Lab. Inc., West Grove, PA, USA) 1:500 dilution).

### Transmission electron microscopy

The organ of Corti was dissected under a dissecting microscope and transferred to a rinse solution (0.18 M sucrose in 0.1 PBS, 3 washes). Tissues were immersed in 1% osmium tetroxide for 2 h. Specimens were dehydrated in increasing alcohol concentrations (50–100%) and embedded in Epon618. Viewing was by contrast phase microscopy, where the sample was implanted in the encasement with the apex of the cochlea upwards and the cochlear axis parallel with the incisal surface. Solidification was achieved by drying in an oven overnight. Embedded samples were placed to the central axis under the anatomical microscope. Semithin sections (1 μm) were prepared with an ultramicrotome (Reichert-Jung, Munich, Germany). Samples were dried at 70–80°C, stained with toluidine blue (1%) and observed for cochlear morphology. Ultrathin sections (50–60 nm) were prepared with an ultramicrotome (Bromma 2088; LKB Produkter, Ontario, Canada). Samples were stained with uracyl-acetate and lead-citrate and images were captured with a Philips CM-120 transmission electron microscope (Philips, Amsterdam, Holland).

## Results

### Hair cell and supporting cell markers show no marked changes

**Figure 1:**
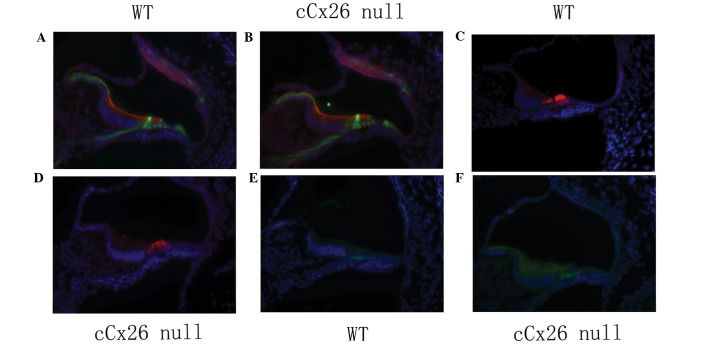
Cellular structures of the organ of Corti at P0 were compared between (A, C and E) WT and (B, D and F) cCx26 null mice. Hair cell and supporting cell specific markers were immunolabeled. All cell nuclei were stained with DAPI (4′,6-Diamidino-2-Phenylindole). (A and B) Supporting cells (pillar cells) were marked with P75 antibody (green fluorescence). Red fluorescent labeling with phalloidin was used to visualize the cell borders. (C and D) Hair cells (including one inner hair cell and three outer hair cells) were labed with mysin6 (red fluorescene). (E and F) Supporting cells were marked with Prox1 antibody (green fluorescence). cCx26 null, conditional connexin 26 knockout mouse; WT, wild type mouse.

Cochlear frozen sections at P3 showed that the organ of Corti and Nuel’s space were not open (Fig. 1A and B)and pillar cells in cochlear-supporting cells had no marked changes between wild-type and mutant mice. (Fig. 1A and B). Prox1 staining of cochlear sertoli cells also showed no marked changes (Fig. 1E and F). The cochlear hair cell marker myosin 6 was not different between cCx26ko and wild-type mice (Fig. 1C and D).

### Tunnel of the organ of Corti and Nuel’s space does not develop prior to hearing onset in cCx26ko mice

**Figure 2:**
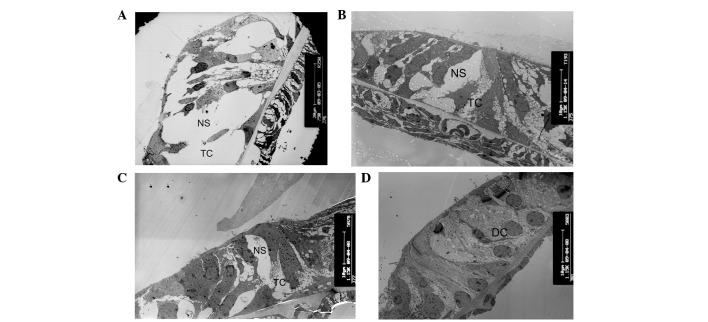
Alterations of tunnel of Corti and Nuel’s space. (A) Tunnel of Corti and Nuel’s space at postnatal day 10 (P10) in wild-type mice (magnification, ×750; apical turn). (B) The tunnel of Corti was not formed at P8 and Nuel’s space was partly formed in cCx26ko mice (magnification, ×1,150; apical turn). (C) The tunnel and Nuel’s space were not formed at P10 in cCx26ko mice (magnification, ×1,150; middle turn). (D) The tunnel of Corti and Nuel’s space at P18 in cCx26ko mice were filled with neighboring enlarged supporting cells (magnification, ×1,150; middle turn). TC, Corti tunnel; NS, Nuel’s space; DC, Deiter’s cell; cCx26ko, conditional connexin 26 knockout.

**Figure 3:**
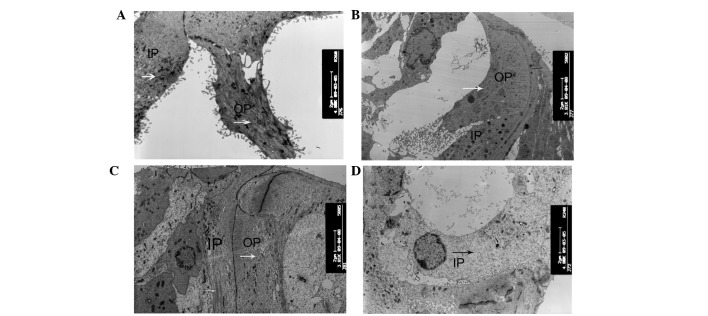
Alterations of microtubules. (A) IPCs and OPCs showed abundant microtubules in P10 wild-type mice (magnification, ×4,800; apical turn). (B and C) Numbers of microtubules in IPCs and OPCs following P10 in cCx26ko mice (magnification, ×3,800; middle turn). (D) Microtubules of IPCs were almost absent in P30 cCx26 mice (magnification, ×4,800; middle turn). IPC, inner pillar cells; OPC, outer pillar cells; arrows, microtubules; cCx26ko, conditional connexin 26 knockout.

The opening of the tunnel of Corti between the inner pillar cells and outer pillar cells was observed at P8, and Nuel’s space formed at P10 in wild-type mice (Fig. 2A). These are important hallmarks in cochlear development. However, the tunnel of Corti was not formed at P8 and Nuel’s space was only partly formed at this postnatal stage in cCx26ko mice (Fig. 2B). As shown in Fig. 2C, the tunnel and Nuel’s space had not yet formed at P10 in cCx26ko mice. The space at the tunnel of Corti and Nuel’s space was occupied by the processes of Deiter’s cells. At P18, the tunnel of Corti and Nuel’s space remained immature in cCx26ko mice. The space was filled with neighboring enlarged supporting cells (Fig. 2D). The changes in microtubules were examined in this region of the organ of Corti, which is significant in the opening of the tunnel and the Nuel’s space. The inner and outer pillar cells showed abundant microtubules in wild-type mice (Fig. 3A). In cCx26ko mice, the numbers of microtubules in the inner and outer pillar cells were reduced following P10 (Fig. 3B) and were even lower at P18 (Fig. 3C). At P30, the microtubules of the inner pillar cells had almost disappeared (Fig. 3D). Thus, the abnormal development of microtubules in pillar cells may be an underlying factor in the inability to generate the opening of the intercellular space between the outer and inner pillar cells.

### Alterations of the cellular ultrastructure in the organ of Corti

**Figure 4:**
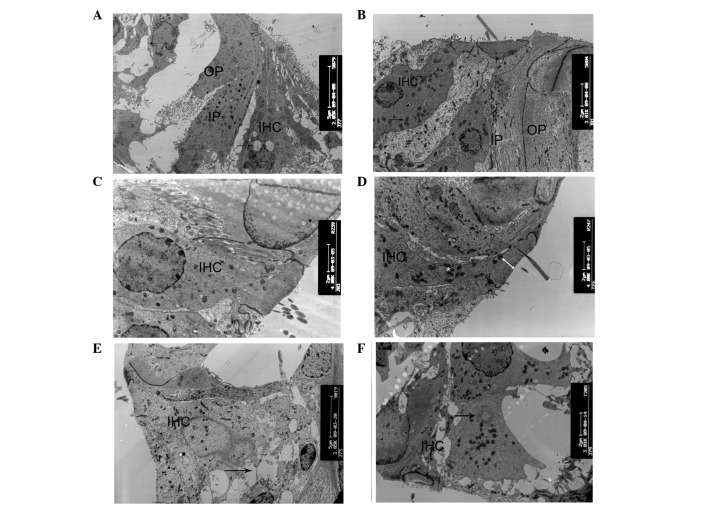
Ultrastructural changes of inner cells. (A) Small number of vacuoles in inner hair cells and intact cell shape in P10 cCx26ko mice (magnification, ×2,850; middle turn). Arrow, vacuoles. (B) Increased number of lysosomes and swollen mitochondria in inner hair cells in P18 cCx26ko mice (magnification, ×3,800; middle turn). Arrows, mitochondria. (C and D) Thinning of dense cuticular plate in inner hair cells at P30 in cCx26ko mice compared with P30 wild-type mice (magnification, ×4,800; middle turn). Arrows, dense cuticular plate. (E and F) Deformed inner hair cells of the basal turn with (E) empty spaces, arrows, from degenerated cells in P60 cCx26ko mice (magnification, ×2,800; basal turn). (F) Arrows, mitochondria with myelin in inner hair cells of P90 cCx26ko mice (magnification, ×2,800, apical turn). IPC, inner pillar cells; OPC, outer pillar cells; IHC, inner hair cells; cCx26ko, conditional connexin 26 knockout.

**Figure 5:**
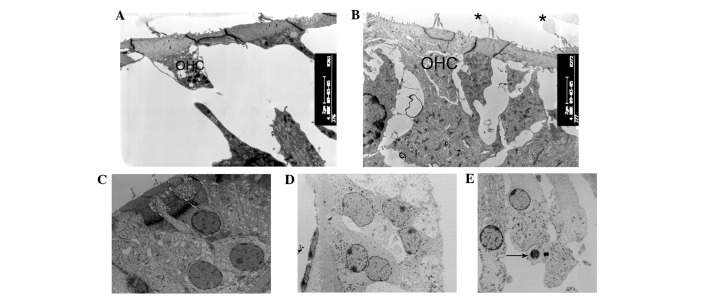
Ultrastructural changes of the outer hair cells regions. (A and B) At postnatal day 10 (P10), the majority of outer hair cells appeared cuboid with enlarged cytoplasm in cCx26ko mice compared with wild-type mice (magnification, ×4,800; middle turn). (C and D) Following P18, outer hair cells became cuboid with unclear cell boundaries and little intercellular space (magnification, ×1,680; middle turn). (E) At P60, nuclei of outer hair cells showed pycknosis and cells were severely degenerated (magnification, ×3,800; basal turn). Arrows, degenerated cells; OHC, outer hair cells; cCx26ko, conditional connexin 26 knockout.

**Figure 6:**
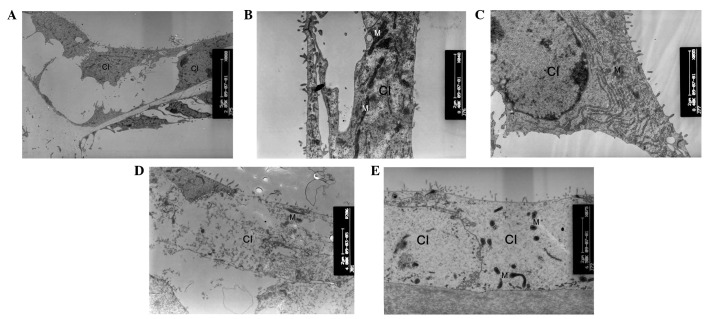
Ultrastructural changes of Claudius cells. (A) At postnatal day 8 (P8), the Claudius cells of cCx26ko mice appeared normal (magnification, ×2,850; apical turn). At P10, (B) mitochondria of wild-type mice were dense and robust (magnification, ×8,400; apical turn) and (C) mitochondria of cCx26ko mice were enlarged (magnification, ×8,400; middle turn). Following P18, (D) Claudius cells degenerated and (E) cytoplasm became scarce and scattered (magnification, ×4,800; middle turn). Fewer mitochondria were observed in Claudius cells at P30 in cCx26ko mice (magnification, ×6,500; middle turn). Cl, Claudius cells; M, mitochondria; cCx26ko, conditional connexin 26 knockout.

**Figure 7:**
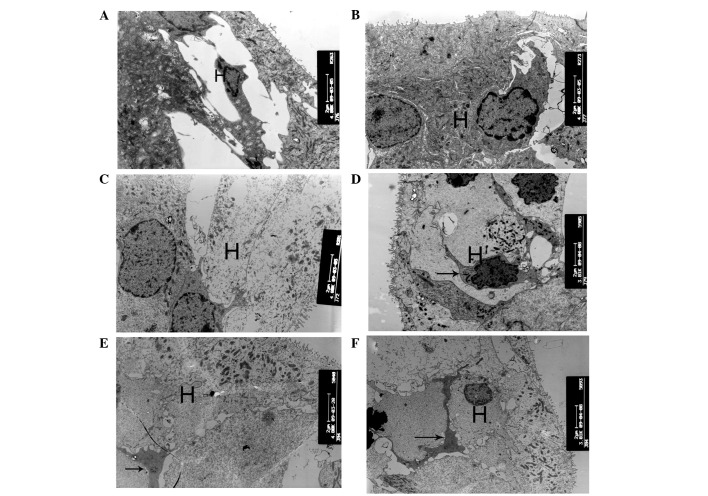
Ultrastructural changes of Hensen cell regions. (A and B) At postnatal day 10 (P10), the Hensen cells of cCx26ko mice had more cytoplasm than P10 wild-type mice (magnification, ×4,800; middle turn). (C) At P30, cytoplasm of Hensen cells was scarce (magnification, ×4,800; middle turn). (D) At P90, cells around the outer hair cell region showed necrosis (magnification, ×3,800; apical turn). Arrows, necrosis. (E and F) Following P180, microglia-like cells appeared in the Hensen cell region at the apical turn (magnification, ×4,800 and ×3,800, respectively; apical turn). Arrows, microglia-like cell. H, Hensen cells; cCx26ko, conditional connexin 26 knockout.

The degeneration process of hair cells and supporting cells was systematically examined at various developmental stages of cCx26ko mice. At P10, only a small number of vacuoles in the inner hair cells were observed (Fig. 4A) and the cell shape was intact. By contrast, at the same developmental stage, the majority of the outer hair cells appeared cuboid with an enlarged cytoplasm and a remaining intercellular gap (Fig. 5A and B). This change was first observed in the Claudius cells at the middle turn. At P8, the Claudius cells of Cx26 knockout mice appeared normal (Fig. 6A). In the Claudius cells at P10, the mitochondria of wild-type mice were dense and robust (Fig. 6B), while the mitochondria of cCx26ko mice appeared to be enlarged (Fig. 6C). Notably, the Hensen cells of cCx26ko mice had more cytoplasm compared with wild-type mice (Fig. 7A and B), indicating an abnormal intracellular metabolism. Following P18, the number of lysosomes increased and mitochondria of the inner hair cells became swollen in the cells of cCx26ko mice (Fig. 4B). The Claudius cells began degenerating, in particular, the cytoplasm became scarce and scattered (Fig. 6D). The dense cuticular plate of the outer hair cells was thinner and the intercellular space had almost disappeared (Fig. 5C). Thinning of dense cuticular plates in inner hair cells occurred at P30 (Fig. 4C and D). At this stage, the outer hair cells became cuboid with unclear cell boundaries (Fig. 5D). The cytoplasm of Hensen cells became scarce (Fig. 7C). In addition, fewer mitochondria were observed in Claudius cells in knockout mice compared with wild-type mice (Fig. 6E).

At P60, the inner hair cells of the basal turn became deformed in cCx26ko mice, with large empty spaces from degenerated cells (Fig. 4E). The nuclei of the outer hair cells showed pyknosis and the cells were severely degenerated (Fig. 5E). At P90, the cells around the outer hair cell region showed signs of necrosis (Fig. 7D) and the majority of other cells in the sensory epithelium at the basal turn had died. At the apical turn, mitochondria with myelin fibers in the inner hair cells were observed (Fig. 4F), indicating damage to the mitochondrial membrane. Following P180, microglia-like cells appeared in the Hensen cell region at the apical turn (Fig. 7E and F), which was the only region in the cochlea where surviving cells were found. The histological features of the apical organ of Corti were anachromasis and enlarged cytoplasm, with pleomorphism with pseudopodia.

These particular cells were first reported in the rat organ of Corti following aminoglycoside ototoxicity (13). Studies showed that these types of cells contain numerous microfilaments and microtubules (14). The cells appear in the Hensen cell region in older stages and were found to be active supportive cells participating in repair and cleanup following damage.

### Ultrastructural changes of the spiral ganglion

**Figure 8:**
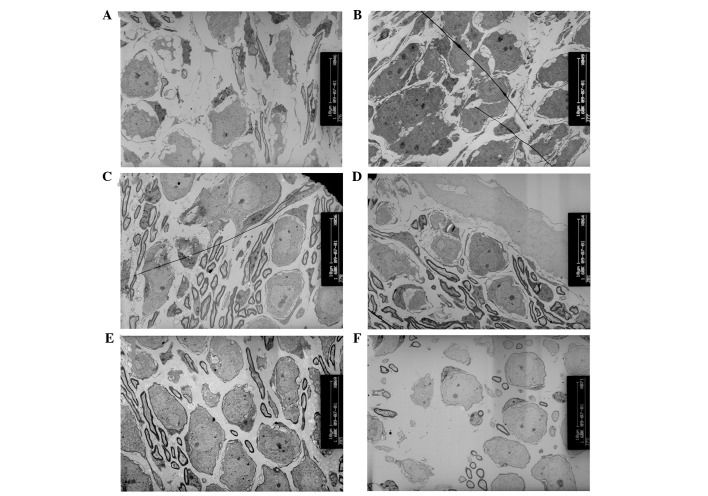
Ultrastructural changes of spiral ganglions. (A–D) No difference in numbers of spiral ganglion neurons were identified at (A and B) P10 (magnification, ×1,600; middle turn) and (C and D) P18 (magnification, ×1,600; middle turn). (E and F) At P30, fewer spiral ganglion neurons were observed in cCx26ko mice compared with wild-type mice (magnification, ×1,600; middle turn). cCx26ko, conditional connexin 26 knockout.

Between P10 and P18, no noticeable differences were observed in the numbers of spiral ganglion neurons (Fig. 8A–D). However, at P30, the numbers of spiral ganglion neurons in cCx26ko mice were distinctively lower compared with Cx26 wild-type mice (Fig. 8E and F). The thickness of the myelin sheath was also found to be decreased in cCx26ko mice.

## Discussion

A decrease in Cx26 protein expression affects the development of structures in the organ of Corti (9), but does not affect the early development of hair cells and supporting cells. No differences were found in markers of hair cells and supporting cells between cCx26ko mice and wild-type mice. The hair cell marker myosin 6, the supporting cell marker prox1 and the pillar cell marker p75 were unchanged. Although the tunnel of Corti was not open in cCx26ko mice, this anomaly was not caused by changes in the three marker proteins. Shim *et al*(15) found that a lack of Sprouty2 leads to ectopic tunnel and tunnel development abnormalities, thus the organ of Corti developmental abnormalities in cCx26ko mice may be associated with the Sprouty2 gene. This hypothesis requires further investigation.

Epithelial gap junctions formed by Cx26 are known to be necessary for normal cochlear functions (16). However, the mechanisms of deafness caused by Cx26 mutations remain unknown. A landmark morphological development immediately prior to the onset of hearing is the opening of the tunnel of Corti and formation of Nuel’s space. The current results are consistent with a study by Inoshita *et al*(17) that observed a different cCx26ko mouse model. Cx26 may directly or indirectly regulate the genes necessary for differentiation of supporting cells at the transcriptional or translational level. Abnormal release of ATP or cell-signaling molecules through Cx26 gap junction hemichannels may also cause the abnormal structure of the pillar cells.

The present observations for the organ of Corti showed that microglia-like cells around the Hensen cells were involved in the cell degeneration process. Following hair cell degeneration, the surrounding supporting cells migrate to fill the spaces left to maintain the integrity of the epithelial cells. Microglia-like cells appeared in later stages of development while the hair cells were undergoing apoptosis. Degeneration is hypothesized to be first initiated by phagocytosis to clear debris or metabolic waste generated by apoptotic cells. The morphological characteristics of irregular protrusions may fill spaces following hair cell damage and form scar-like tissue (14).

GJs are widely hypothesized to connect supporting cells in the organ of Corti, primarily to provide ionic pathways for rapid removal of K^+^ around the base of hair cells. However, the precise function of GJs in the cochlea remains unknown. A working hypothesis explaining hair cell apoptosis is the K^+^ recycling theory (18). A previous study showed that K^+^ recirculation in the cochlea may be affected by the level and properties of GJ intercellular communication (19). Stimulation of hair cells by sound generates an increase in extracellular K^+^, which transports into outer hair cells and then into Deiters’ cells and adjacent supporting cells by the K-Cl co-transporter Kcc4, as well as extracellular signals, including ATP-induced IP3 production and Ca^2+^ release from the ER compartment. Ca^2+^ may activate the Cl^−^ channels of supporting cells, allowing Cl^−^ to move to the extracellular side, favoring K^+^ transport to the endolymph. The absence of Cx26 blocks GJ-mediated K^+^ recycling, which is driven by transduction events around the inner hair cells. This model predicts that cellular pathological changes in the organ of Corti of cCx26ko mice should begin at a site close to the inner hair cells. However, the pathological changes were observed to occur initially around the outer hair cells. Specifically, Claudius cells were the first to degenerate. Wang *et al*(9) reported first observing cell degeneration in Claudius cells at ~P8. In the current study, swollen mitochondria were initially observed in Claudius cells at ~P10 in the middle turn cochlea. High metabolism in cochlear cells may produce reactive oxygen species (ROS), regulated by an antioxidative enzyme system. ROS are negative regulators of GJs, reducing intercellular coupling. Ablation of Cx26 in the cochlea may increase the accumulation of the ROS in the cochlea, resulting in mitochondrial swelling and an increase in lysosome numbers. Swollen mitochondria were observed in numerous cells types in the organ of Corti, implying that abnormal energy metabolism may be correlated with a decrease in ATP synthesis. In addition, mitochondria are involved in cellular apoptosis (20), accelerating the progress of degeneration and necrosis. Apoptosis of hair cells is hypothesized to cause metabolic stress and energy deficiency.

The current results support the hypothesis that a number of pathological changes in the organ of Corti of cCx26 mice occur prior to the formation of the endocochlear potential and prior to the onset of hearing, when endocochlear potential is low and K^+^ recycling is lacking. The timing of these changes indicates that the basis for deafness in Cx26 mutant mice is not a lack of K^+^ recycling.
